# The role of TIM-3 in sepsis: a promising target for immunotherapy?

**DOI:** 10.3389/fimmu.2024.1328667

**Published:** 2024-03-21

**Authors:** Changli Wang, Jinhai Liu, Qi Wu, Zhi Wang, Baoji Hu, Lulong Bo

**Affiliations:** ^1^ Faculty of Anesthesiology, Changhai Hospital, Naval Medical University, Shanghai, China; ^2^ Department of Anesthesiology, Shanghai Pudong Hospital, Fudan University Pudong Medical Center, Shanghai, China

**Keywords:** TIM-3, Sepsis, immunotherapy, inflammatory response, therapeutic target

## Abstract

Sepsis remains a significant cause of mortality and morbidity worldwide, with limited effective treatment options. The T-cell immunoglobulin and mucin domain-containing molecule 3 (TIM-3) has emerged as a potential therapeutic target in various immune-related disorders. This narrative review aims to explore the role of TIM-3 in sepsis and evaluate its potential as a promising target for immunotherapy. We discuss the dynamic expression patterns of TIM-3 during sepsis and its involvement in regulating immune responses. Furthermore, we examine the preclinical studies investigating the regulation of TIM-3 signaling pathways in septic models, highlighting the potential therapeutic benefits and challenges associated with targeting TIM-3. Overall, this review emphasizes the importance of TIM-3 in sepsis pathogenesis and underscores the promising prospects of TIM-3-based immunotherapy as a potential strategy to combat this life-threatening condition.

## 1 Introduction

Sepsis, a potentially fatal medical condition resulting from an uncontrolled immune response to infection, remains a significant public health challenge (1). Its clinical urgency is highlighted by its staggering morbidity and mortality rates, thus making it one of the primary causes of death globally with millions of cases reported each year (2). During sepsis, overactivation of the immune system leads to systemic tissue damage and organ failure, amplifying its devastating impact (3). To address this complex immunopathology, identification of effective therapeutic targets is essential (4). T-cell Immunoglobulin and Mucin-domain containing-3 (TIM-3), due to its critical role in steering immune responses across several disease contexts, has emerged as a potential target (5–7). Initially identified on T cells, TIM-3 is now known to be expressed on multiple immune cell types, including natural killer cells, dendritic cells, and macrophages (8–10). Altered expression and dysregulation of TIM-3 have been observed in sepsis patients, hinting at its involvement in the pathogenesis of this condition (11–13).

Immunotherapy, which aims to modulate the immune response and restore immune homeostasis, carries potential promise for sepsis treatment (14). Among various immunotherapeutic strategies being investigated, inhibiting or modulating TIM-3 holds significant potential (15). Preliminary preclinical studies focusing on TIM-3 blockade or targeting have shown promising results, further motivating investigations into its mechanisms of action in sepsis (12, 16–18).

This review aims to provide a comprehensive exploration of the role of TIM-3 in sepsis and its potential as a therapeutic target. We will delve into the expression patterns, regulatory mechanisms, and interactions of TIM-3 during sepsis to shed light on its contribution to the pathogenesis of this condition. Additionally, by dissecting the mechanisms through which TIM-3 acts and understanding the challenges it presents, we aim to guide the field towards innovative strategies that harness TIM-3’s therapeutic potential in sepsis.

## 2 TIM-3: an overview

**Figure 1:**
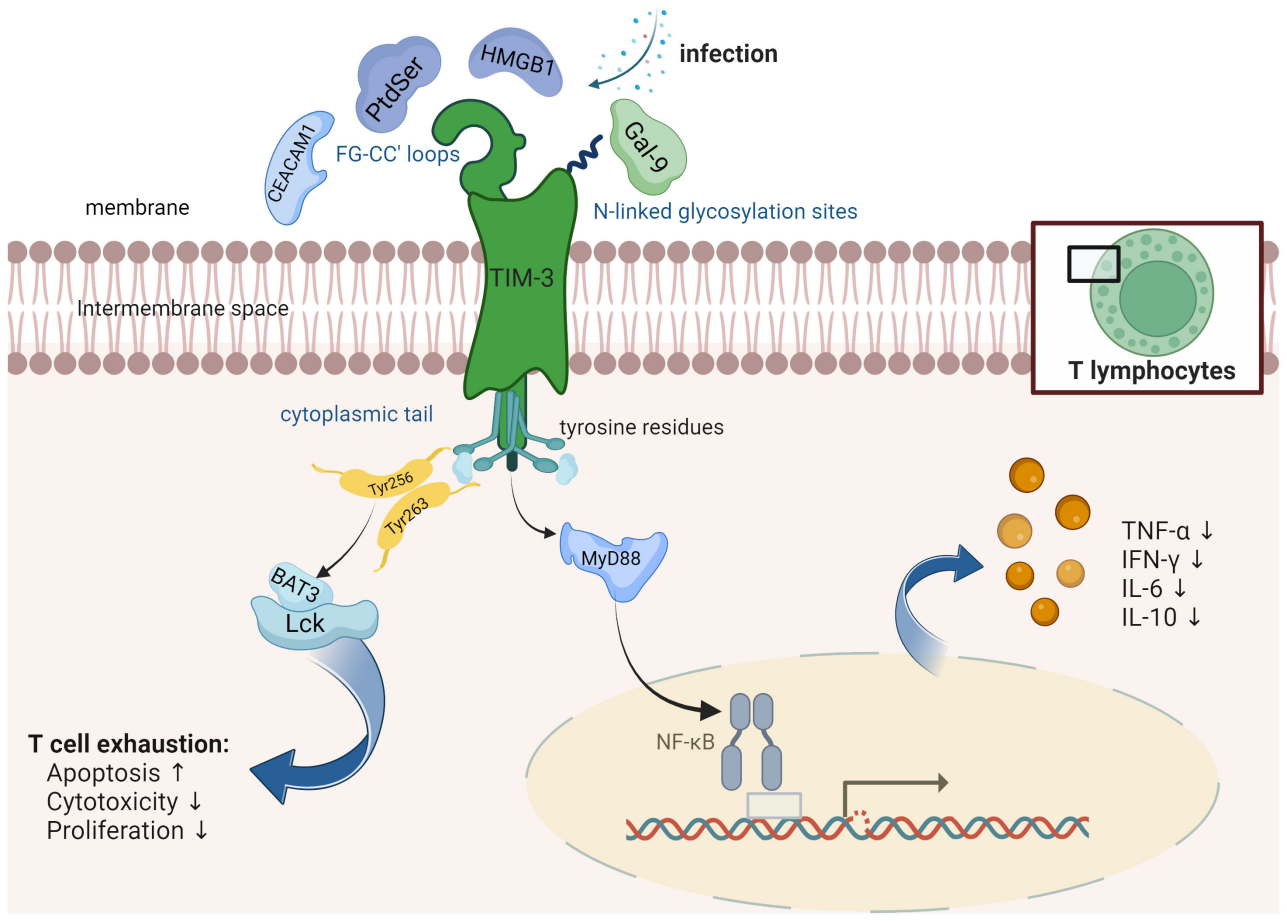
TIM-3 is a protein that consists of several distinct structural elements, namely, an extracellular immunoglobulin variable (IgV) domain, a mucin stalk featuring N- and O-linked glycosylation sites, and an intracellular tail containing conserved tyrosine residues. It is found to be expressed on various immune cells including T cells, natural killer (NK) cells, and antigen-presenting cells (APCs). TIM-3 interacts with different receptors such as Phosphatidylserine (PtdSer), carcinoembryonic antigen-related cell adhesion molecule 1 (CEACAM1), and high-mobility group box 1 (HMGB1). These receptors bind to specific regions known as the FG-CC’ loops on TIM-3. Additionally, Galectin-9 (Gal-9), a distinct protein, binds specifically to the N-linked glycosylation sites on TIM-3. TIM-3 plays a significant role in modulating the NF-κB pathway in the context of infections, thereby exerting control over the production and release of cytokines. Human Leukocyte Antigen-B-associated transcript 3 (BAT3) is an essential adaptor protein known for its interactions with the cytoplasmic domain of TIM-3. BAT3 plays a critical role in the regulation of T cell exhaustion, a state characterized by impaired effector function and increased expression of inhibitory receptors.

TIM-3 is a transmembrane protein and an integral part of the TIM family that comprises eight members, including TIM-1, TIM-3, and TIM-4 in humans (19). The human TIM-3 gene is located on chromosome 5q33.2, consisting of 1116 nucleotides and encoding 302 amino acids (20). The mouse TIM-3 gene is located on chromosome 11B1.1, encoding 281 amino acids. Human TIM-3 has 63% homology with mouse TIM-3. This protein includes three domains: the extracellular domain, the transmembrane domain, and the intracellular domain. The extracellular domain has an N-terminal immunoglobulin variable (IgV) domain with FG-CC’ loops and N-linked glycosylation sites, a mucin-like domain with O-linked glycosylation sites, and a stalk domain with N-linked glycosylation (19) (**Figure 1**). The transmembrane domain spans across the cell membrane, whereas the intracellular domain holds a cytoplasmic tail with five tyrosine residues. The IgV domain contains the binding site for its ligands such as Phosphatidylserine (PtdSer), carcinoembryonic antigen-related cell adhesion molecule 1 (CEACAM1), and high-mobility group box 1 (HMGB1), all of which bind to the FG-CC’ loops. Galectin-9 (Gal-9), on the other hand, binds to the N-linked glycosylation (21).

The function of TIM-3 is determined by its context, and it can act both as an activating and inhibitory receptor (10). It interacts with four primary ligands: Gal-9 (22), CEACAM1 (23), HMGB1 (24), and PtdSer (25) (**Figure 1**). All four ligands engage with the IgV domain of TIM-3, GAL-9 being the predominant one. When Gal-9 binds to TIM-3, it prompts apoptosis in T cells and minimizes T cell responses (22). In CD8^+^ T cells, co-expression of TIM-3 with other inhibitory immune checkpoint molecules such as programmed cell death protein 1 (PD-1) and CD160, along with stimulatory molecules like 2B4 and lymphocyte activation gene-3 (LAG-3), is linked to T cell differentiation, activation, and increase in IFN-γ and TNF-α levels (26, 27), upon T cell receptor (TCR) stimulation, suggesting a potential role in T cell activation and effector function. Furthermore, blocking the TIM-3 pathway could potentially inhibit Treg activation. These intricate interactions and regulatory mechanisms underscore the multifaceted role of TIM-3 in controlling immune responses (28). On the other hand, TIM-3 has also been implicated in T cell exhaustion. Persistent antigen exposure leads to sustained TIM-3 expression on T cells, contributing to T cell dysfunction and exhaustion characterized by impaired proliferation and cytokine production. It is now established that the amino acid residues Tyr256 and Tyr263 play a crucial role in facilitating interactions between Human Leukocyte Antigen-B-associated transcript 3 (BAT3) and the tyrosine kinase FYN (29). Phosphorylation of these residues leads to the dissociation of BAT3 from TIM-3, enabling TIM-3 to carry out its inhibitory function (30). Additionally, FYN has the potential to modulate TIM-3-mediated inhibitory signaling by competitively binding to the same region on TIM-3 as BAT3, suggesting a possible competition between FYN and BAT3 for TIM-3 binding.

TIM-3 plays an instrumental role in regulating immune responses by striking a balance between immune activation and tolerance. Through its negative regulatory function, TIM-3 helps avert excessive or prolonged immune activation, which could lead to tissue damage and autoimmunity (31). In T cells, engagement of TIM-3 inhibits signaling pathways mediated by the TCR, resulting in reduced T cell proliferation, cytokine production, and effector functions (32). This regulatory role of TIM-3 is further supported by its ability to induce T cell exhaustion (29). Treg cells, which are crucial for maintaining immune homeostasis and preventing autoimmunity, have been shown to upregulate TIM-3 expression in certain inflammatory environments. TIM-3-expressing Treg cells may exhibit enhanced suppressive function and contribute to the regulation of immune responses by dampening excessive inflammation and promoting tolerance.

TIM-3’s regulatory effects are not confined solely to T cells; they extend beyond, impacting other immune cells (29). For instance, TIM-3 signaling hinders the cytotoxicity of natural killer cells agains viral infections (33). Myeloid cells, such as dendritic cells and macrophages, can also express TIM-3 and play a role in modulating immune responses. Within dendritic cells, TIM-3 engagement results in diminished antigen presentation and impaired T cell priming (8). TIM-3 expression on myeloid cells has been associated with the suppression of T cell activation and the promotion of immune tolerance (34). Moreover, interactions between TIM-3-expressing myeloid cells and TIM-3-expressing T cells may further regulate immune responses in a complex manner. NK cells, as innate immune effectors, can also express TIM-3. TIM-3 expression on NK cells has been associated with functional impairment and diminished cytotoxic activity. The engagement of TIM-3 on NK cells by its ligands may contribute to the regulation of NK cell function and impact overall immune responses (13). These observations suggest that TIM-3 serves as a vital modulator of immune responses across various cell types, contributing significantly to immune homeostasis and preventing excessive immunopathology (29). Recent studies have provided compelling evidence suggesting that TIM-3 operates as a co-stimulatory receptor, rather than solely as an inhibitory one, in the activation of T cells in specific contexts (35, 36). This dual role of TIM-3 is crucial for the optimal functioning of T cell immunity, as it facilitates the generation of short-lived effector T cells while simultaneously suppressing the development of memory precursors (37). Consequently, TIM-3 exhibits pleiotropic effects in the maintenance of immune homeostasis, exerting its regulatory functions by modulating various immune cell populations in a cell-type- and context-specific manner.

It is now established that TIM-3 exhibits two distinct functionalities. On one hand, it serves as a crucial physiological inhibitor of inflammatory T cell responses, particularly in the context of autoimmune conditions. Conversely, it also plays a detrimental role as a promoter of T cell dysfunction and exhaustion in cancer and chronic viral infections (35). Despite these well-documented roles, there remains a critical inquiry into the underlying mechanisms that dictate the divergent cell fates of TIM-3^+^ cells. Specifically, the question persists as to whether these distinct functionalities are dictated by external factors influencing T cells, or if they are a result of differential signaling pathways downstream of TIM-3. BAT3 is an essential adaptor protein known for its interaction with the cytoplasmic domain of TIM-3. Recent investigations have shed light on the pivotal role of BAT3 as a molecular checkpoint in regulating T cell exhaustion. Specifically, studies have demonstrated that the absence of BAT3 leads to a pronounced exhaustion phenotype in T cells, dampening their ability to mediate neuroinflammation driven by autoreactive T cells (38). In preclinical models of autoimmunity, such as experimental autoimmune encephalomyelitis, and cancer, the deficiency of BAT3 in dendritic cells has been shown to significantly alter the T cell landscape. This alteration manifests as a decrease in Th1, Th17, and cytotoxic effector cells, alongside an increase in regulatory T cells and exhausted CD8^+^ tumor-infiltrating lymphocytes. Consequently, this immune remodeling results in the amelioration of autoimmune responses and the facilitation of tumor progression (39).

In conclusion, TIM-3 emerges as a versatile immune regulatory molecule with a significant role in orchestrating immune responses across different cell types. Through its expression patterns and interactions with ligands, TIM-3 wields both activating and inhibitory influences on immune cells, thus playing an instrumental role in maintaining immune homeostasis. However, a comprehensive understanding of the intricate mechanisms and potential therapeutic applications of targeting TIM-3 in diverse disease settings necessitates further, more exhaustive studies. Moreover, according to the current research on TIM-3, there are still many controversial aspects, such as the identification of relevant ligands, specific downstream signaling pathways, which still need to be further studied.

## 3 The role of TIM-3 in sepsis

Sepsis is known to induce upregulation of TIM-3 expression on various immune cells, including T cells, natural killer (NK) cells, dendritic cells (DCs), and macrophages (8, 9, 40). Sepsis is a dysregulated systemic host response to infection that can lead to organ dysfunction and is associated with high morbidity and mortality. In sepsis, TIM-3 expression on immune cells, particularly T cells and myeloid cells, has been linked to the development of immune paralysis and dysfunction, contributing to the immunosuppressive state observed in septic patients (41). TIM-3 expression can be induced rapidly in response to the overwhelming inflammatory and infectious stimuli. This upregulation of TIM-3 expression has been the subject of several studies investigating its role in sepsis, which have shed light on its potential as a target for immunotherapy. In a prospective study conducted by Boomer et al., a cohort of 24 patients diagnosed with severe sepsis within 24 hours of its onset was examined to investigate the expression of inhibitory receptors on lymphocytes, specifically those associated with cell exhaustion. The study revealed a significant upregulation of cytotoxic T lymphocyte antigen-4 (CTLA-4), TIM-3, and LAG-3 receptors on T lymphocytes in individuals with acute sepsis. This notable upregulation of exhaustion-associated receptors potentially contributes to the immune-suppressed state frequently observed in severe sepsis cases. Consequently, the study suggests that therapeutic interventions aimed at reversing T cell exhaustion could hold promising potential in restoring immune functionality and enhancing survival rates among sepsis patients (17). However, Spec et al. found no significant difference in TIM-3 expression on CD4^+^ and CD8^+^ T cells during Candida sepsis among critically ill patients compared to controls (42). In patients with sepsis-induced immunosuppression, Huang et al. observed a significant elevation in TIM-3 expression on CD4^+^ T cells, and the percentage of TIM-3^+^ CD4^+^ T cells correlated with sepsis-induced immunosuppression mortality (18). Furthermore, blocking the TIM-3 signaling pathway was shown to promote the release of IL-10 and TNF-α by T lymphocytes in septic patients (12). Moreover, in sepsis, the interactions of TIM-3 with its ligands, such as Galectin-9, HMGB1, or CEACAM1, may play a crucial role in modulating immune responses and influencing the outcome of sepsis (18). The engagement of TIM-3 in sepsis may lead to the functional impairment of T cells and myeloid cells, exacerbating the dysregulated immune response and contributing to immunosuppression. Understanding the dynamics of TIM-3 signaling pathways in sepsis could provide novel insights into potential therapeutic strategies for modulating immune function and improving outcomes in septic patients.

In addition to its significant effect on CD4^+^T cells in sepsis, many studies have found that TIM-3 also plays an important role in the immune response of other types of T cells. In a study by Yan et al., they investigated sepsis-induced acute respiratory distress syndrome (ARDS) and found a positive correlation between the proportion of TIM-3^+^ CD8^+^ T cells and the duration of shock. They also observed that non-survivors had significantly higher expression of both PD-1 and TIM-3 on CD8^+^ T cells compared to survivors (43). Another study explored the relationship between CD8^+^ T cell exhaustion and ARDS in sepsis patients (44). The study revealed that decreased CD8^+^ T cell counts and proliferation rates were associated with non-survival in ARDS patients. Additionally, increased expression of inhibitory receptors PD-1 and TIM-3 was related to worse organ function and longer shock duration, respectively. These findings suggest that CD8^+^ T cells and coinhibitory receptors can serve as independent prognostic markers for sepsis-induced ARDS. Therefore, targeting TIM-3 could hold potential for immunotherapy in sepsis. Furthermore, in a mouse cecal ligation and puncture (CLP) model and human septic patients, TIM-3 was highly upregulated in liver CD8^+^ T cells. The expression of TIM-3 in liver CD8^+^ T cells displayed a biphasic pattern, and its deletion resulted in reduced CD8^+^ T cell apoptosis. Administration of α-lactose, a molecule similar in structure to Gal-9, reduced TIM-3 expression and liver injury in sepsis. These findings suggest that targeting TIM-3 to enhance CD8^+^ T cell immune response may improve outcomes in septic patients (45). Yuan et al. reported a significant reduction in the proportion of Vδ1T cells in septic patients compared to healthy controls, which was correlated with disease severity (46). Additionally, septic patients exhibited elevated expression of immunosuppressive molecules such as glucocorticoid-induced tumor necrosis factor receptor (GITR) (47), CTLA-4 (48), and TIM-3 on Vδ1T cells. This increase in immunosuppressive markers on Vδ1T cells correlated with T cell proliferation inhibition and impaired interferon secretion, indicating a higher degree of immunosuppression in sepsis. Wu et al. discovered a close correlation between the expression of TIM-3 and the functional status of NKT cells in septic patients (13). They found that upregulated TIM-3 expression promoted NKT cell activation and apoptosis during early sepsis, leading to worse disease severity and prognosis. To investigate the potential of targeting TIM-3 as an immunomodulatory strategy for sepsis management, they blocked the TIM-3/Galectin-9 signal axis using α-lactose in a mouse model of CLP. This inhibition of NKT cell apoptosis protected against septic challenge, indicating the potential of targeting TIM-3 in sepsis (13). Similarly, Yao et al. demonstrated the upregulation of TIM-3 in NKT cells, which mediated NKT cell apoptosis in both mouse septic models and human septic patients (49).

TIM-3 is expressed in a variety of immune cells which is involved in pathogen killing or antigen presentation of these immune cells in sepsis. A previous study also found that an increased number of suppressive monocytes (PDL1^+ve^ and TIM-3^+ve^) at baseline could identify patients with acute-on-chronic liver failure (ACLF) who are at high risk of developing sepsis within 48-72 hours of hospitalization. Additionally, ex vivo LPS-stimulated monocytes from patients with ACLF and sepsis showed a significant increase in the expression of PD-L1 and TIM-3 (50). Ren et al. reported elevated TIM-3 expression on monocytes in sepsis patients compared to severe sepsis, septic shock, and control patients. Soluble TIM-3 (sTIM-3) levels in the plasma of the septic shock group were higher than those of the sepsis or severe sepsis groups, and sTIM-3 levels correlated with eventual non-survivors (51). These findings suggest that TIM-3 expression on monocytes and sTIM-3 exhibit distinct profiles among patients with varying severity of sepsis, highlighting the need for mechanistic studies to elucidate the exact role of TIM-3 during sepsis. However, in a study by Yang et al., TIM-3 mRNA expression in peripheral blood mononuclear cells was significantly lower in severe sepsis patients compared to sepsis patients. This downregulation of TIM-3 correlated with increased levels of C-reactive protein, a clinical marker of inflammatory status (52). Hou et al. observed a dynamic inverse correlation between TIM-3 expression and IFN-γ production in NK cells from LPS-induced septic mice (53). The expression of TIM-3 on NK cells significantly increased at 24 hours after LPS injection, with a moderate increase at 4 hours but declining to undetectable levels by 12 hours. *In vitro* experiments showed that blocking the TIM-3 pathway increased IFN-γ production and decreased apoptosis of NK cells. These findings suggest that the TIM-3 pathway plays an inhibitory role in NK cell function and may be a potential target for modulating the excessive inflammatory response in LPS-induced endotoxic shock (53).

A recent prospective study in Germany involving 712 sepsis patients from three ICUs investigated the functional single-nucleotide polymorphisms (SNPs) of TIM-3 (54). The results demonstrated a lower 28-day mortality in patients with the TIM-3 rs1036199 AA homozygous genotype and TIM-3 rs10515746 CC homozygous genotype, compared to carriers of the C-allele and A-allele, respectively. Additionally, patients with the rs1036199 AA genotype had more Gram-positive and *Staphylococcus epidermidis* infections, while rs10515746 CC homozygotes had more *Staphylococcus epidermidis* infections. These identified TIM-3 genetic variants may serve as prognostic markers and aid in identifying high-risk septic patients. Clinical studies registered on clinicaltrials.gov were also retrieved. Three studies on the relationship between TIM-3 and sepsis were found all from China. One observational study (NCT02180009) specifically examined the expression of TIM-3 on monocytes and its soluble expression in septic patients. Another observational study (NCT01801839) aimed to investigate the imbalance of anti- and pro-inflammation in septic patients by analyzing antigen presentation, cytokine secretion, and TIM-3 expression on monocytes/macrophages. The third observational study (NCT02319876) focused on the expression of TIM-3 on lymphocytes in sepsis, considering that immunosuppression is a major cause of death in septic patients. Unfortunately, there have been no further updates on these projects on the website, such as completion status or publication of related literature, to provide corresponding results. Despite the lack of published results, researchers are actively pursuing the clinical study of TIM-3 in sepsis due to its potential clinical significance.

The involvement of TIM-3 in sepsis pathogenesis is complex and multifaceted. On one hand, TIM-3 has been identified as an inhibitory receptor with immunosuppressive effects, promoting immune tolerance and dampening excessive inflammation. Such immunosuppressive function may contribute to immune paralysis in sepsis, resulting in impaired host defense against invading pathogens. Therefore, understanding the delicate balance between these dual roles of TIM-3 necessitates further investigation supported by the latest research findings. In addition to its direct influence on immune cells, TIM-3 reciprocally interacts with other immune checkpoints in sepsis, forming complex regulatory networks. Accumulating evidence suggests that the interaction between TIM-3 and PD-1 synergistically suppresses T cell responses, promoting T cell exhaustion in sepsis (55). Furthermore, the co-expression of TIM-3 and CTLA-4 has been associated with suboptimal T cell function and worse clinical outcomes in sepsis patients (56). These interactions underscore the importance of comprehensive studies on multiple immune inhibitory pathways in sepsis, paving the way for novel combination immunotherapy strategies.

## 4 TIM-3 as a therapeutic target in sepsis

**Figure 2:**
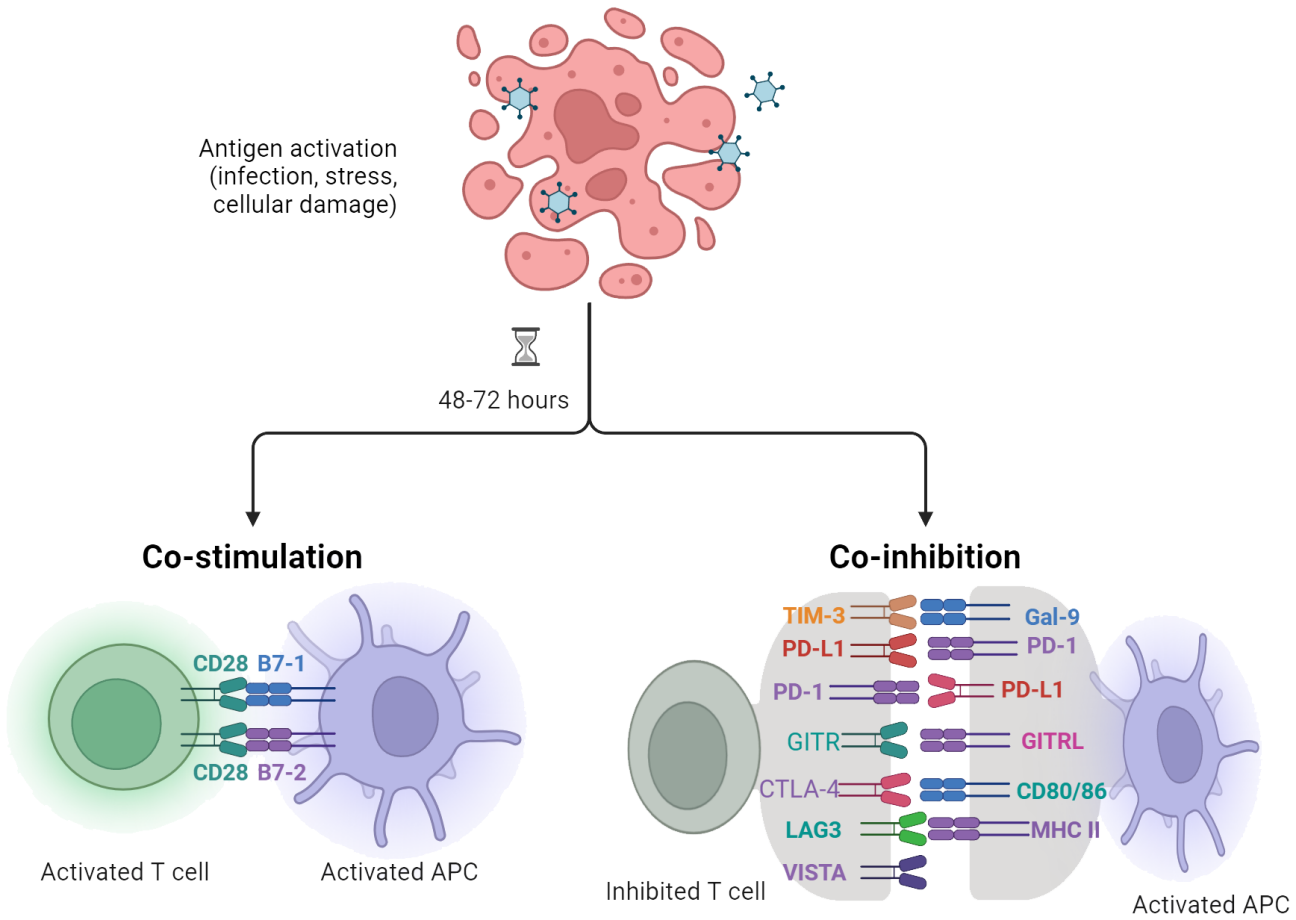
Antigen-presenting cells (APCs), including dendritic cells (DCs), play a crucial role in modulating T cell responses specific to infection, stress, or cellular injury. The regulation and activation of T lymphocytes are contingent upon signaling mediated by the T cell receptor (TCR) as well as cosignaling receptors, which transmit negative (–) or positive (+) signals.

Current therapeutic strategies for sepsis primarily focus on support-based approaches, such as antibiotics, fluid resuscitation, and organ support (57–59). However, there is an urgent need for specific immunotherapies that can target and modulate the excessive immune response associated with sepsis to prevent organ damage. This highlights the importance of exploring new treatment options that can restore immune homeostasis in septic patients. Immunomodulatory targets, particularly immune checkpoints, have been extensively studied in sepsis (60). Immune checkpoints are molecules involved in regulating immune function, and their inhibitors have shown success in enhancing anti-tumor immune responses in cancer treatment (61). This raises the question of whether targeting immune checkpoints could also provide benefits for sepsis patients (**Figure 2**).

In septic episodes, the presence of inflammatory cytokines and danger signals triggers an upregulation of TIM-3 expression in various immune cells, including T cells, macrophages, and dendritic cells. This upregulation of TIM-3 signaling has been highlighted as significant in promoting immune dysfunction and exhaustion, suggesting its potential role in sepsis progression (62, 63). Hypothetically, therapeutic targeting of TIM-3 might rejuvenate immune cell function, thereby improving clinical outcomes in sepsis patients. Preclinical studies using animal models of sepsis have provided support for this hypothesis, demonstrating encouraging results when interventions blocking or modulating TIM-3 signaling were employed. These interventions resulted in improved survival rates, reduced inflammation, and decreased organ dysfunction among septic animals (60).

For instance, in our previous study, we found that TIM-3 expression is increased in spleen CD8^+^ T cells of mice during CLP-induced sepsis. Blocking TIM-3 with anti-TIM-3 antibodies at the early stage of sepsis reduced its severity, alleviated lung and liver injuries, decreased inflammatory responses, and prevented lymphocyte apopsosis (64). These findings indicate that anti-TIM-3 antibodies may serve as a promising immunotherapy target for sepsis. Furthermore, Xia et al. discovered that blockade of the TIM-3 signaling pathway with TIM-3 antibody contributed to a significant elevation of IL-10 and TNF-α in the supernatant of T lymphocytes in septic patients. Blocking both TIM-3 and PD-1 induced the positivity of IL-10- and TNF-α-expressing cells in peripheral monocytes. Significant changes were noticed in the expression of TIM-3 and PD-1 in both T lymphocytes and monocytes. Blocking TIM-3 and PD-1 improved the function of lymphocytes and monocytes, suggesting their crucial roles in the immune response during sepsis (12).

Moreover, Wang et al. observed an increase in the expression of TIM-3 on CD4^+^ T cells, CD8^+^ T cells, and NK cells in the peritoneal lavage 24 hours after a single LPS injection (65). The expression of TIM-3 on splenic NK cells was also significantly elevated. This upregulation of TIM-3 served as a marker of immune exhaustion, with TIM-3^+^ T cells and NK cells exhibiting reduced IFN-γ production. In septic mice, blockade of the TIM-3 pathway hastened mortality, while its activation prolonged survival. Furthermore, the administration of a TIM-3 blocking antibody *in vitro* restored IFN-γ release from splenocytes, reduced splenocyte apoptosis, and increased levels of IFN-γ and TNF-α in septic mice. Conversely, activation of the TIM-3 pathway inhibited cell proliferation. These findings indicate that the TIM-3 signaling pathway plays a crucial role as a negative mediator in LPS-induced endotoxic shock and could serve as a promising therapeutic target for sepsis (65). However, some studies have found that a single LPS injection did not increase PD-1 and TIM-3 expression in CD4^+^ T and CD8^+^ T cells in mice, whereas recurrent sepsis induced by multiple LPS stimulations resulted in a significant increase in PD-1 and TIM-3 expression (66).

Liu et al. conducted a study using a murine model of CLP sepsis to investigate the impact of long-term exposure to glucocorticoids on CD4^+^ T cells and the cytokine storm in sepsis (67). Their findings revealed that chronic exposure to glucocorticoids exacerbated apoptosis of CD4^+^ T cells and the cytokine storm. This exacerbation was mechanistically linked to the increase in CD3^+^ TIM-3^+^ T cells. These CD3^+^ TIM-3^+^ T cells were found to express high levels of multiple cytokine genes during infections, indicating a significant role of TIM-3 in regulating T cell biology. Furthermore, *in vitro* studies demonstrated that anti-TIM-3 treatment enhanced the inflammatory activity of CD3^+^ T cells. Thus, this study established a causal relationship between chronic exposure to glucocorticoids and an excessive inflammatory response mediated by T cells during infections, partly driven by dysregulation of CD3^+^ TIM-3^+^ T cells.

Kadowaki et al. investigated the protective effects of Gal-9 in a murine model of sepsis induced by CLP (68). To demonstrate these effects, they utilized Gal-9 transgenic mice and administered therapeutic Gal-9. The results showed that Gal-9 reduced levels of TNF-α, IL-6, IL-10, and HMGB1, while increasing levels of IL-15 and IL-17 in both plasma and spleen. Moreover, Gal-9 increased the frequencies of NKT cells and PDCA-1^+^ CD11c^+^ macrophages (pDC-like macrophages), while decreasing the frequency of TIM-3^+^ CD4^+^ T cells, particularly Th1 and Th17 cells. Based on these findings, it can be inferred that Gal-9 exerts therapeutic effects on polymicrobial sepsis by expanding NKT cells and pDC-like macrophages, as well as modulating the production of early and late proinflammatory cytokines.

In a study by Luo et al., the combination of mesenchymal stem cells (MSCs) with Gal-9 was found to improve survival rates and kidney function in mice with sepsis-associated acute kidney injury (SA-AKI) (69). This treatment not only reduced inflammation but also restored balance to the Th17/Treg cell ratio and enhanced the expression of anti-inflammatory factors. Interestingly, when the Gal-9/TIM-3 pathway was blocked using soluble TIM-3, the therapeutic effects of MSCs were reversed, resulting in kidney injury and increased mortality. These findings suggest that targeting TIM-3 could be a promising approach for immunotherapy in sepsis. Additionally, the knockdown of Gal-9 in MSCs using small interfering RNA hindered the therapeutic effect of MSCs. Similarly, the blockade of the Gal-9/TIM-3 pathway using α-lactose or anti-TIM-3 inhibited the induction of Tregs and suppressed the differentiation of Th17 cells when MSCs were cocultured with CD4^+^ T cells (70). These results indicate that the beneficial effect of MSCs against SA-AKI may be partially mediated by the induction of Tregs and inhibition of Th17 cells through the Gal-9/TIM-3 pathway. However, another studyverified that Gal-9 is not a ligand for TIM-3 on CD4^+^ T cells during sepsis (18). Instead, their results indicated that TIM-3 exhibits a relatively strong binding affinity for HMGB1, suggesting its potential as a receptor for HMGB1 in TIM-3^+^ CD4^+^ T cells during sepsis. Therefore, further research is needed to investigate the role of Gal-9 in sepsis and whether it exerts its effects by regulating TIM-3.

Zhao et al. conducted a study to investigate the role of TIM-3 signaling in sepsis using a CLP model. Their findings demonstrated that blocking TIM-3 signaling with soluble TIM-3-Immunoglobulin (sTIM-3-IgG) during the acute phase of sepsis exacerbated macrophage pro-inflammatory responses and lymphocyte apoptosis (71). However, during the late phase of sepsis, it enhanced the anti-inflammatory phenotype of macrophages and CD4^+^ T cells. These results indicate a dual role for TIM-3 in the regulation of sepsis. Moreover, mice over-expressing TIM-3 exhibited attenuated sepsis-induced immunosuppression and improved survival. Similar results were observed when administering the TIM-3 ligand Gal-9. In contrast, Yang et al. showed that blocking TIM-3 signaling using an anti-TIM-3 antibody or sTIM-3-IgG increased sepsis severity and decreased survival in a CLP model. This study also revealed the negative regulatory role of TIM-3 on TLR4-mediated responses of macrophages, leading to the inhibition of macrophage activation. These findings suggest that the TLR4 signaling pathway plays a crucial role in TIM-3-related immune homeostatic mechanisms during sepsis (52).

Furthermore, Huang et al. demonstrated that the conditional deletion of TIM-3 in CD4^+^ T cells, as well as systemic TIM-3 deletion, reduced mortality in mice with sepsis by preserving organ function. They found that TIM-3^+^ CD4^+^ T cells had reduced proliferative ability and elevated expression of inhibitory markers compared to Tim-3^-^ CD4^+^ T cells. Additionally, their results indicated that blocking TIM-3 in CD4^+^ T cells led to the activation of the NF-κB/TNF-α signaling pathway, which counteracted sepsis-induced immunosuppression (18). Similarly, Yang et al. revealed that TIM-3 plays a role in maintaining sepsis by negatively regulating LPS-TLR4-mediated NF-κB activation. In their study, blockade and/or downregulation of TIM-3 was associated with increased severity of sepsis (52).

In summary, pre-clinical studies investigating the role of TIM-3 in sepsis have produced inconsistent results. Blocking TIM-3 signaling during the acute phase of sepsis aggravated the pro-inflammatory response and lymphocyte apoptosis, but it enhanced the anti-inflammatory phenotype during the late phase. Mice over-expressing TIM-3 displayed improved survival and attenuated immunosuppression, while blocking TIM-3 signaling increased sepsis severity and decreased survival. These findings provide additional insights into the biological mechanisms of TIM-3, suggesting that the TIM-3 signaling pathways may play distinct regulatory roles on T cell and macrophage function in a manner that is dependent on the disease stage and microbial activity. Given the dynamic and potentially life-threatening nature of sepsis as a rapidly progressing condition, there is a clear need for further research to fully comprehend the mechanisms underlying TIM-3’s dual role in sepsis. Moreover, investigating the interplay between TIM-3 and other immune checkpoint molecules, such as PD-1 and CTLA-4, may yield valuable insights into potential combination therapies for sepsis. Additionally, exploring the impact of TIM-3 on various immune cell populations, including T cells, macrophages, and DCs, could provide a better understanding of its diverse effects on the immune response during sepsis. Ultimately, a comprehensive understanding of the role of TIM-3 and other immune checkpoint molecules in the pathophysiology of sepsis is essential for the development of innovative therapeutic strategies aimed at enhancing outcomes for sepsis patients.

## 5 Challenges and future perspectives

Targeting TIM-3 in sepsis holds potential, but the path forward is filled with complex challenges. A major limitation is the dynamic nature of the immune response during sepsis (60). Immune responses are not uniform and vary based on the stage of sepsis and the specific cell types involved (72). The expression and regulation of TIM-3 may differ across the stages of sepsis and between different cell types like monocytes and T cells, contributing to heterogeneity that complicates therapy development. This complexity presents a challenge to both clinicians and researchers, underscoring the need for a more nuanced understanding of TIM-3’s roles within these varied contexts.

Another hurdle is the possible off-target effects when manipulating TIM-3. As TIM-3 is expressed on non-immune cells such as endothelial cells, therapeutic intervention may unintentionally impact the functions of these cells, potentially causing unwanted effects like vascular permeability changes or even microvascular thrombosis (73). Therefore, carefully delineating potential off-target effects is vital when developing TIM-3-based therapies.

Furthermore, it is essential to determine the optimal timing and duration for TIM-3 modulation. Sepsis is time-sensitive, and interventions at varying disease stages could yield divergent outcomes. Premature or delayed intervention might worsen the disease, miss the therapeutic window, or lead to unexpected side effects (74). Hence, identifying critical intervention points where TIM-3 targeting would be most beneficial is essential. Moreover, strategies for timed delivery of these therapies should be a priority.

Given these limitations and challenges, several strategies can be explored. First, further research is required to elucidate the precise mechanisms of TIM-3 activation and its interactions with other immune checkpoints during sepsis. Understanding these molecular pathways will offer insights into how to effectively manipulate TIM-3 without causing undesired side effects. Second, the development of selective TIM-3 inhibitors or agonists could help minimize off-target effects. By specifically targeting immune cells expressing TIM-3 while sparing non-immune cells, we could achieve a more precise modulation of the immune response in sepsis, reducing potential adverse effects. A third approach involves combination therapies targeting multiple immune checkpoints concurrently. Given that sepsis involves dysregulated immune responses, targeting a single molecule may not fully restore immune homeostasis (75). Combining TIM-3 blockade or modulation with other immunotherapies, such as PD-1/PD-L1 inhibitors or TLR agonists, may amplify therapeutic outcomes, assuming no unforeseen antagonistic interactions occur (76).

By addressing these challenges, exploring potential strategies, and focusing on future research directions, TIM-3-targeted immunotherapy in sepsis shows considerable promise. Considering the high stakes associated with sepsis treatment, advancement in this area is an urgent need. Continued efforts in this domain will unquestionably contribute to creating effective treatments for this life-threatening condition, thereby improving patient outcomes in the future.

## 6 Conclusion

In conclusion, the growing comprehension of TIM-3’s role in sepsis offers insightful perspectives on the complex immune dysregulation characterizing this life-threatening condition. The distinct expression patterns and regulatory mechanisms of TIM-3 during sepsis not only indicate its crucial function in disease pathogenesis but also highlight its potential as a promising target for therapeutic intervention. By modulating the signaling pathways associated with TIM-3 activation, we may be better equipped to impact various responses including immune cell functions, inflammation levels, tissue damage, and organ dysfunction in sepsis. As our understanding of TIM-3 continues to expand, it appears as a hopeful prospect for effective immunotherapy in sepsis management.

## Author contributions

CW: Writing – original draft, Investigation, Conceptualization. JL: Writing – original draft, Methodology, Investigation. QW: Writing – original draft, Methodology, Investigation, Data curation. ZW: Writing – original draft, Resources, Methodology, Data curation. BH: Writing – review & editing, Supervision, Investigation, Conceptualization, Resources. LB: Funding acquisition, Writing – review & editing, Supervision, Conceptualization.

## References

[1] Singer M Deutschman CS Seymour CW Shankar-Hari M Annane D Bauer M. The third international consensus definitions for sepsis and septic shock (Sepsis-3). JAMA. (2016) 315:801–10. doi: 10.1001/jama.2016.0287 PMC4968574 26903338

[2] Rudd KE Johnson SC Agesa KM Shackelford KA Tsoi D Kievlan DR. Global, regional, and national sepsis incidence and mortality, 1990-2017: Analysis for the global burden of disease study. Lancet. (2020) 395:200–11. doi: 10.1016/S0140-6736(19)32989-7 PMC6970225 31954465

[3] van der Poll T Shankar-Hari M Wiersinga WJ. The immunology of sepsis. Immunity. (2021) 54:2450–64. doi: 10.1016/j.immuni.2021.10.012 34758337

[4] Zhang YY Ning BT. Signaling pathways and intervention therapies in sepsis. Signal Transduct Target Ther. (2021) 6:407. doi: 10.1038/s41392-021-00816-9 34824200 PMC8613465

[5] Chiba M Yanaba K Hayashi M Yoshihara Y Nakagawa H. Clinical significance of serum soluble T-cell immunoglobulin and mucin domain 3 levels in systemic sclerosis: Association with disease severity. J Dermatol. (2017) 44:194–7. doi: 10.1111/1346-8138.13610 27651303

[6] Zilber E Martin GE Willberg CB Fox J Nwokolo N Fidler S. Soluble plasma programmed death 1 (Pd-1) and tim-3 in primary hiv infection. AIDS. (2019) 33:1253–6. doi: 10.1097/QAD.0000000000002165 31045943

[7] Goncalves Silva I Yasinska IM Sakhnevych SS Fiedler W Wellbrock J Bardelli M. The tim-3-galectin-9 secretory pathway is involved in the immune escape of human acute myeloid leukemia cells. EBioMedicine. (2017) 22:44–57. doi: 10.1016/j.ebiom.2017.07.018 28750861 PMC5552242

[8] Anderson AC Anderson DE Bregoli L Hastings WD Kassam N Lei C. Promotion of tissue inflammation by the immune receptor tim-3 expressed on innate immune cells. Science. (2007) 318:1141–3. doi: 10.1126/science.1148536 18006747

[9] Monney L Sabatos CA Gaglia JL Ryu A Waldner H Chernova T. Th1-specific cell surface protein tim-3 regulates macrophage activation and severity of an autoimmune disease. Nature. (2002) 415:536–41. doi: 10.1038/415536a 11823861

[10] Rezaei M Tan J Zeng C Li Y Ganjalikhani-Hakemi M. Tim-3 in leukemia; immune response and beyond. Front Oncol. (2021) 11:753677. doi: 10.3389/fonc.2021.753677 34660319 PMC8514831

[11] Patil NK Guo Y Luan L Sherwood ER. Targeting immune cell checkpoints during sepsis. Int J Mol Sci. (2017) 18(11):2413. doi: 10.3390/ijms18112413 29135922 PMC5713381

[12] Xia Q Wei L Zhang Y Sheng J Wu W Zhang Y. Immune checkpoint receptors tim-3 and pd-1 regulate monocyte and T lymphocyte function in septic patients. Mediators Inflammation. (2018) 2018:1632902. doi: 10.1155/2018/1632902 PMC6282152 30595665

[13] Wu H Tang T Deng H Chen D Zhang C Luo J. Immune checkpoint molecule tim-3 promotes nkt cell apoptosis and predicts poorer prognosis in sepsis. Clin Immunol. (2023) 254:109249. doi: 10.1016/j.clim.2023.109249 36736642

[14] Torres LK Pickkers P van der Poll T. Sepsis-induced immunosuppression. Annu Rev Physiol. (2022) 84:157–81. doi: 10.1146/annurev-physiol-061121-040214 34705481

[15] Rienzo M Skirecki T Monneret G Timsit JF. Immune checkpoint inhibitors for the treatment of sepsis:Insights from preclinical and clinical development. Expert Opin Investig Drugs. (2022) 31:885–94. doi: 10.1080/13543784.2022.2102477 35944174

[16] Venet F Monneret G. Advances in the understanding and treatment of sepsis-induced immunosuppression. Nat Rev Nephrol. (2018) 14:121–37. doi: 10.1038/nrneph.2017.165 29225343

[17] Boomer JS Shuherk-Shaffer J Hotchkiss RS Green JM. A prospective analysis of lymphocyte phenotype and function over the course of acute sepsis. Crit Care. (2012) 16:R112. doi: 10.1186/cc11404 22742734 PMC3580670

[18] Huang S Liu D Sun J Zhang H Zhang J Wang Q. Tim-3 regulates sepsis-induced immunosuppression by inhibiting the nf-kappab signaling pathway in cd4 T cells. Mol Ther. (2022) 30:1227–38. doi: 10.1016/j.ymthe.2021.12.013 PMC8899604 34933101

[19] Umetsu DT Umetsu SE Freeman GJ DeKruyff RH. Tim gene family and their role in atopic diseases. Curr Top Microbiol Immunol. (2008) 321:201–15. doi: 10.1007/978-3-540-75203-5_10 18727494

[20] McIntire JJ Umetsu SE Akbari O Potter M Kuchroo VK Barsh GS. Identification of tapr (an airway hyperreactivity regulatory locus) and the linked tim gene family. Nat Immunol. (2001) 2:1109–16. doi: 10.1038/ni739 11725301

[21] Kandel S Adhikary P Li G Cheng K. The tim3/gal9 signaling pathway: An emerging target for cancer immunotherapy. Cancer Lett. (2021) 510:67–78. doi: 10.1016/j.canlet.2021.04.011 33895262 PMC8168453

[22] Zhu C Anderson AC Schubart A Xiong H Imitola J Khoury SJ. The tim-3 ligand galectin-9 negatively regulates T helper type 1 immunity. Nat Immunol. (2005) 6:1245–52. doi: 10.1038/ni1271 16286920

[23] Huang YH Zhu C Kondo Y Anderson AC Gandhi A Russell A. Ceacam1 regulates tim-3-mediated tolerance and exhaustion. Nature. (2015) 517:386–90. doi: 10.1038/nature13848 PMC4297519 25363763

[24] Chiba S Baghdadi M Akiba H Yoshiyama H Kinoshita I Dosaka-Akita H. Tumor-infiltrating dcs suppress nucleic acid-mediated innate immune responses through interactions between the receptor tim-3 and the alarmin hmgb1. Nat Immunol. (2012) 13:832–42. doi: 10.1038/ni.2376 PMC3622453 22842346

[25] DeKruyff RH Bu X Ballesteros A Santiago C Chim YL Lee HH. T cell/transmembrane, ig, and mucin-3 allelic variants differentially recognize phosphatidylserine and mediate phagocytosis of apoptotic cells. J Immunol. (2010) 184:1918–30. doi: 10.4049/jimmunol.0903059 PMC3128800 20083673

[26] Ozkazanc D Yoyen-Ermis D Tavukcuoglu E Buyukasik Y Esendagli G. Functional exhaustion of cd4(+) T cells induced by co-stimulatory signals from myeloid leukaemia cells. Immunology. (2016) 149:460–71. doi: 10.1111/imm.12665 PMC5095494 27565576

[27] Gorman JV Starbeck-Miller G Pham NL Traver GL Rothman PB Harty JT. Tim-3 directly enhances cd8 T cell responses to acute listeria monocytogenes infection. J Immunol. (2014) 192:3133–42. doi: 10.4049/jimmunol.1302290 PMC3965639 24567532

[28] Anderson AC Joller N Kuchroo VK. Lag-3, tim-3, and tigit: Co-inhibitory receptors with specialized functions in immune regulation. Immunity. (2016) 44:989–1004. doi: 10.1016/j.immuni.2016.05.001 27192565 PMC4942846

[29] Wolf Y Anderson AC Kuchroo VK. Tim3 comes of age as an inhibitory receptor. Nat Rev Immunol. (2020) 20:173–85. doi: 10.1038/s41577-019-0224-6 PMC7327798 31676858

[30] Rangachari M Zhu C Sakuishi K Xiao S Karman J Chen A. Bat3 promotes T cell responses and autoimmunity by repressing tim-3-mediated cell death and exhaustion. Nat Med. (2012) 18:1394–400. doi: 10.1038/nm.2871 PMC3491118 22863785

[31] Joller N Kuchroo VK. Tim-3, lag-3, and tigit. Curr Top Microbiol Immunol. (2017) 410:127–56. doi: 10.1007/82_2017_62 PMC5902028 28900677

[32] Schultheiss C Paschold L Simnica D Mohme M Willscher E von Wenserski L. Next-generation sequencing of T and B cell receptor repertoires from covid-19 patients showed signatures associated with severity of disease. Immunity. (2020) 53:442–55.e4. doi: 10.1016/j.immuni.2020.06.024 32668194 PMC7324317

[33] Ndhlovu LC Lopez-Verges S Barbour JD Jones RB Jha AR Long BR. Tim-3 marks human natural killer cell maturation and suppresses cell-mediated cytotoxicity. Blood. (2012) 119:3734–43. doi: 10.1182/blood-2011-11-392951 PMC3335380 22383801

[34] Qin S Xu L Yi M Yu S Wu K Luo S. Novel immune checkpoint targets: Moving beyond pd-1 and ctla-4. Mol Cancer. (2019) 18:155. doi: 10.1186/s12943-019-1091-2 31690319 PMC6833286

[35] Tang R Rangachari M Kuchroo VK. Tim-3: A co-receptor with diverse roles in T cell exhaustion and tolerance. Semin Immunol. (2019) 42:101302. doi: 10.1016/j.smim.2019.101302 31604535

[36] Lu C Chen H Wang C Yang F Li J Liu H. An emerging role of tim3 expression on T cells in chronic kidney inflammation. Front Immunol. (2021) 12:798683. doi: 10.3389/fimmu.2021.798683 35154075 PMC8825483

[37] Avery L Filderman J Szymczak-Workman AL Kane LP. Tim-3 co-stimulation promotes short-lived effector T cells, restricts memory precursors, and is dispensable for T cell exhaustion. Proc Natl Acad Sci U.S.A. (2018) 115:2455–60. doi: 10.1073/pnas.1712107115 PMC5877951 29463725

[38] Zhu C Dixon KO Newcomer K Gu G Xiao S Zaghouani S. Tim-3 adaptor protein bat3 is a molecular checkpoint of T cell terminal differentiation and exhaustion. Sci Adv. (2021) 7(18):eabd2710. doi: 10.1126/sciadv.abd2710 33931442 PMC8087420

[39] Tang R Acharya N Subramanian A Purohit V Tabaka M Hou Y. Tim-3 adapter protein bat3 acts as an endogenous regulator of tolerogenic dendritic cell function. Sci Immunol. (2022) 7:eabm0631. doi: 10.1126/sciimmunol.abm0631 35275752 PMC9273260

[40] Chen H Zha J Tang R Chen G. T-cell immunoglobulin and mucin-domain containing-3 (Tim-3): Solving a key puzzle in autoimmune diseases. Int Immunopharmacol. (2023) 121:110418. doi: 10.1016/j.intimp.2023.110418 37290326

[41] Liu D Huang SY Sun JH Zhang HC Cai QL Gao C. Sepsis-induced immunosuppression: Mechanisms, diagnosis and current treatment options. Mil Med Res. (2022) 9:56. doi: 10.1186/s40779-022-00422-y 36209190 PMC9547753

[42] Spec A Shindo Y Burnham CA Wilson S Ablordeppey EA Beiter ER. T cells from patients with candida sepsis display a suppressive immunophenotype. Crit Care. (2016) 20:15. doi: 10.1186/s13054-016-1182-z 26786705 PMC4719210

[43] Yan L Chen Y Han Y Tong C. Role of cd8(+) T cell exhaustion in the progression and prognosis of acute respiratory distress syndrome induced by sepsis: A prospective observational study. BMC Emerg Med. (2022) 22:182. doi: 10.1186/s12873-022-00733-2 36402952 PMC9675152

[44] Ni L Cheng ML Feng Y Zhao H Liu J Ye F. Impaired cellular immunity to Sars-Cov-2 in severe Covid-19 patients. Front Immunol. (2021) 12:603563. doi: 10.3389/fimmu.2021.603563 33603759 PMC7884325

[45] Wei Z Li P Yao Y Deng H Yi S Zhang C. Alpha-lactose reverses liver injury *via* blockade of tim-3-mediated cd8 apoptosis in sepsis. Clin Immunol. (2018) 192:78–84. doi: 10.1016/j.clim.2018.04.010 29689313

[46] Yuan F Yin H Tan J Zheng K Mei X Yuan L. The proportion of vdelta1t cells in peripheral blood correlated with prognosis of sepsis. Iran J Immunol. (2022) 19:232–42. doi: 10.22034/iji.2022.89256.1934 36190378

[47] Scumpia PO Delano MJ Kelly-Scumpia KM Weinstein JS Wynn JL Winfield RD. Treatment with gitr agonistic antibody corrects adaptive immune dysfunction in sepsis. Blood. (2007) 110:3673–81. doi: 10.1182/blood-2007-04-087171 PMC2077315 17690255

[48] Mewes C Buttner B Hinz J Alpert A Popov AF Ghadimi M. Ctla-4 genetic variants predict survival in patients with sepsis. J Clin Med. (2019) 8(1):70. doi: 10.3390/jcm8010070 30634576 PMC6352177

[49] Yao Y Deng H Li P Zhang J Zhang J Wang D. Alpha-lactose improves the survival of septic mice by blockade of tim-3 signaling to prevent nkt cell apoptosis and attenuate cytokine storm. Shock. (2017) 47:337–45. doi: 10.1097/SHK.0000000000000717 27504802

[50] Yadav P Trehanpati N Maiwall R Sehgal R Singh R Islam M. Soluble factors and suppressive monocytes can predict early development of sepsis in acute-on-chronic liver failure. Hepatol Commun. (2022) 6:2105–20. doi: 10.1002/hep4.1949 PMC9315131 35502507

[51] Ren F Li J Jiang X Xiao K Zhang D Zhao Z. Plasma soluble tim-3 emerges as an inhibitor in sepsis: Sepsis contrary to membrane tim-3 on monocytes. Tissue Antigens. (2015) 86:325–32. doi: 10.1111/tan.12653 26373631

[52] Yang X Jiang X Chen G Xiao Y Geng S Kang C. T cell ig mucin-3 promotes homeostasis of sepsis by negatively regulating the tlr response. J Immunol. (2013) 190:2068–79. doi: 10.4049/jimmunol.1202661 23365080

[53] Hou H Liu W Wu S Lu Y Peng J Zhu Y. Tim-3 negatively mediates natural killer cell function in lps-induced endotoxic shock. PLoS One. (2014) 9:e110585. doi: 10.1371/journal.pone.0110585 25337993 PMC4206431

[54] Mewes C Alexander T Buttner B Hinz J Alpert A Popov AF. Tim-3 genetic variants are associated with altered clinical outcome and susceptibility to gram-positive infections in patients with sepsis. Int J Mol Sci. (2020) 21(21):8318. doi: 10.3390/ijms21218318 33171904 PMC7664272

[55] Yang R Sun L Li CF Wang YH Yao J Li H. Galectin-9 interacts with pd-1 and tim-3 to regulate T cell death and is a target for cancer immunotherapy. Nat Commun. (2021) 12:832. doi: 10.1038/s41467-021-21099-2 33547304 PMC7864927

[56] Sorkhabi AD Sarkesh A Fotouhi A Saeedi H Aghebati-Maleki L. Cancer combination therapies by silencing of ctla-4, pd-L1, and tim3 in osteosarcoma. IUBMB Life. (2022) 74:908–17. doi: 10.1002/iub.2655 35638098

[57] Dellinger RP Levy MM Rhodes A Annane D Gerlach H Opal SM. Surviving sepsis campaign: International guidelines for management of severe sepsis and septic shock: 2012. Crit Care Med. (2013) 41:580–637. doi: 10.1097/CCM.0b013e31827e83af 23353941

[58] Blanco J Muriel-Bombin A Sagredo V Taboada F Gandia F Tamayo L. Incidence, organ dysfunction and mortality in severe sepsis: A spanish multicentre study. Crit Care. (2008) 12:R158. doi: 10.1186/cc7157 19091069 PMC2646323

[59] Hu B Ji W Bo L Bian J. How to improve the care of septic patients following "Surviving sepsis campaign: International guidelines for management of sepsis and septic shock 2021"? J Intensive Med. (2023) 3:144–6. doi: 10.1016/j.jointm.2022.08.001 PMC10175702 37188122

[60] Salomao R Ferreira BL Salomao MC Santos SS Azevedo LCP Brunialti MKC. Sepsis: Evolving concepts and challenges. Braz J Med Biol Res. (2019) 52:e8595. doi: 10.1590/1414-431X20198595 30994733 PMC6472937

[61] Pearce EL Pearce EJ. Metabolic pathways in immune cell activation and quiescence. Immunity. (2013) 38:633–43. doi: 10.1016/j.immuni.2013.04.005 PMC3654249 23601682

[62] Calvano SE Xiao W Richards DR Felciano RM Baker HV Cho RJ. A network-based analysis of systemic inflammation in humans. Nature. (2005) 437:1032–7. doi: 10.1038/nature03985 16136080

[63] Rello J Valenzuela-Sanchez F Ruiz-Rodriguez M Moyano S. Sepsis: A review of advances in management. Adv Ther. (2017) 34:2393–411. doi: 10.1007/s12325-017-0622-8 PMC5702377 29022217

[64] Liu S Wang C Jiang Z Deng X Bo L. Tim-3 blockade decreases the apoptosis of cd8(+) T cells and reduces the severity of sepsis in mice. J Surg Res. (2022) 279:8–16. doi: 10.1016/j.jss.2022.05.014 35716447

[65] Wang F Hou H Xu L Jane M Peng J Lu Y. Tim-3 signaling pathway as a novel negative mediator in lipopolysaccharide-induced endotoxic shock. Hum Immunol. (2014) 75:470–8. doi: 10.1016/j.humimm.2014.02.001 24561184

[66] He W Xiao K Xu J Guan W Xie S Wang K. Recurrent sepsis exacerbates cd4(+) T cell exhaustion and decreases antiviral immune responses. Front Immunol. (2021) 12:627435. doi: 10.3389/fimmu.2021.627435 33717146 PMC7946831

[67] Liu Z Chen H Tan C Zha J Liu H Chen G. Activation of cd3 + Tim3 + T cells contributes to excessive inflammatory response during glucocorticoid treatment. Biochem Pharmacol. (2023) 212:115551. doi: 10.1016/j.bcp.2023.115551 37044297

[68] Kadowaki T Morishita A Niki T Hara J Sato M Tani J. Galectin-9 prolongs the survival of septic mice by expanding tim-3-expressing natural killer T cells and pdca-1+ Cd11c+ Macrophages. Crit Care. (2013) 17:R284. doi: 10.1186/cc13147 24321251 PMC4056346

[69] Luo C Luo F Che L Zhang H Zhao L Zhang W. Mesenchymal stem cells protect against sepsis-associated acute kidney injury by inducing gal-9/tim-3 to remodel immune homeostasis. Ren Fail. (2023) 45:2187229. doi: 10.1080/0886022X.2023.2187229 36883358 PMC10013538

[70] Luo C Luo F Man X Liu X Zhao L Che L. Mesenchymal stem cells attenuate sepsis-associated acute kidney injury by changing the balance of th17 cells/tregs *via* gal-9/tim-3. Curr Stem Cell Res Ther. (2023) 18:540–50. doi: 10.2174/1574888X17666220511151343 35546754

[71] Zhao Z Jiang X Kang C Xiao Y Hou C Yu J. Blockade of the T cell immunoglobulin and mucin domain protein 3 pathway exacerbates sepsis-induced immune deviation and immunosuppression. Clin Exp Immunol. (2014) 178:279–91. doi: 10.1111/cei.12401 PMC4233378 24945079

[72] Biswas SK Lopez-Collazo E. Endotoxin tolerance: New mechanisms, molecules and clinical significance. Trends Immunol. (2009) 30:475–87. doi: 10.1016/j.it.2009.07.009 19781994

[73] Cong Y Wang X Wang S Qiao G Li Y Cao J. Tim-3 promotes tube formation and decreases tight junction formation in vascular endothelial cells. Biosci Rep. (2020) 40(10):BSR20202130. doi: 10.1042/BSR20202130 33015716 PMC7560514

[74] Srzic I Nesek Adam V Tunjic Pejak D. Sepsis definition: What's new in the treatment guidelines. Acta Clin Croat. (2022) 61:67–72. doi: 10.20471/acc.2022.61.s1.11 PMC9536156 36304809

[75] Faix JD. Biomarkers of sepsis. Crit Rev Clin Lab Sci. (2013) 50:23–36. doi: 10.3109/10408363.2013.764490 23480440 PMC3613962

[76] Nakamori Y Park EJ Shimaoka M. Immune deregulation in sepsis and septic shock: Reversing immune paralysis by targeting pd-1/pd-L1 pathway. Front Immunol. (2020) 11:624279. doi: 10.3389/fimmu.2020.624279 33679715 PMC7925640

